# History of childhood abuse is associated with less positive treatment outcomes in socially stable women with alcohol use disorder

**DOI:** 10.1186/s12905-019-0857-4

**Published:** 2019-12-12

**Authors:** Fides Schückher, Tabita Sellin, Ingemar Engström, Kristina Berglund

**Affiliations:** 1 University Health Care Research Center, Faculty of Medicine and Health, Örebro University, SE 701 82 Örebro, Sweden; 2 Department of Psychology, University of Gothenburg, Göteborg, Sweden

**Keywords:** Adult women, Alcohol use disorder, Childhood abuse, Treatment outcome

## Abstract

**Background:**

To examine the relationship between treatment outcome, as measured according to change in alcohol consumption, and a history of childhood abuse (emotional, physical, sexual) in socially stable women undergoing treatment for alcohol use disorder (AUD).

**Methods:**

Participants were assessed using the Addiction Severity Index and the Mini International Neuropsychiatric Interview at the beginning of treatment (*n* = 75), end of treatment (*n* = 59) and 12 month follow-up after treatment (*n* = 57). Self-report data on alcohol consumption were obtained at all three time-points using the Alcohol Habits Inventory-Revised 2. Self-report data on childhood maltreatment were obtained at the beginning of treatment using the Childhood Trauma Questionnaire-short form. Study outcomes were changes in alcohol consumption (grams of pure alcohol per week), risk-drinking and reported abstinence.

**Results:**

Of the 75 women enrolled, 38 (50.7%) reported a history of childhood abuse and the rest did not. Both groups showed a significant improvement in all three outcomes at the end of treatment and at 12-month follow-up. At the end of treatment, a significant inter-group difference was found for reported abstinence (non-abused group, 39.3% vs abused, 12.9%; *p* < 0.05). At 12-month follow-up, significant inter-group differences were observed for all treatment outcomes, with superior outcomes being found for the non-abused group, including a higher proportion of women with reported abstinence (55.6% vs 13.3%; *p* < 0.01).

**Conclusion:**

The present findings suggest that an evaluation of a possible history of childhood abuse is warranted in all women seeking treatment for AUD, irrespective of social stability. In terms of clinical practice, the results suggest that additional interventions may be warranted in this population.

## Background

Alcohol use disorder (AUD) affects both men and women, and is associated with severe impairments in physical and mental health in all groups of society worldwide [1]. Research has identified several risk factors for both AUD development and treatment outcome. Data from a limited number of studies suggest that treatment outcome may be influenced by a history of childhood abuse. This study will therefore try to broaden our knowledge of the association between childhood abuse and treatment outcome in women with AUD.

A commonly applied definition of childhood abuse is words or overt actions that are deliberate or intentional by a parent or other caregiver that cause harm, potential harm, or threat to harm a child, even if harm is not the intended consequence [2]. In general, childhood abuse comprises the following three forms: physical, sexual and emotional (or psychological) abuse [2, 3]. In the field of AUD, the most widely investigated forms of childhood abuse are sexual and physical abuse [4–6]. However, recent research into risk factors for AUD has placed increased emphasis on emotional abuse [7, 8].

Childhood abuse is associated with increased psychopathology, including major depression [5, 9], bipolar disorder [10], anxiety disorder [5], attempted suicide [5, 9], post-traumatic stress disorder [5, 9], AUD and substance use disorder (SUD) [5, 9, 11, 12]. Childhood abuse has been associated with an earlier age of AUD onset [4, 13, 14], greater AUD severity [8, 15], and an increased risk for persistent alcohol dependence in both sexes [16]. Research has shown that, in women, childhood sexual and physical abuse is an important risk factor for the development of AUD [12]. In women with a history of childhood abuse and neglect, research also has demonstrated an association between independent stressful events and heavier drinking density compared with women not exposed [17], and a faster transition from non-problematic drinking to risk-drinking [6]. In a study with a mixed–sex cohort the reported prevalence of childhood emotional abuse was 47.5%, physical abuse 38.9% and for sexual abuse 21.1% in participants with AUD [8].

Although the available literature on predictors of AUD treatment outcome is relatively extensive [18–20], few studies to date have examined the association between childhood abuse and treatment outcome in women with AUD. In a study of men and women undergoing inpatient alcohol treatment that involved survival analyses, an association was found between poorer drinking outcomes, such as shorter time to first drink and relapse after treatment, and a history of childhood sexual abuse. By contrast, the authors found no association with a history of childhood physical abuse [21].

Other studies have examined the effect of childhood abuse on treatment outcome in participants undergoing treatment for addiction disorders. A study of homeless women undergoing residential treatment for SUD found worse psychological and social functioning outcomes in subjects with a history of childhood abuse (sexual, physical and/or emotional) [22]. In a mixed-sex cohort of participants with SUD seeking treatment, Pirard et al. (2005) showed that subjects with a history of childhood sexual and/or physical abuse tended to engage in alcohol and polydrug use, while non-abused subjects preferred heroin and cocaine. Over a 12-month follow-up period, abused subjects, showed worse psychiatric outcomes than did the non-abused group, but there were no significant differences in terms of outcomes for alcohol or drug use severity [23]. Charney et al. (2007) examined a mixed-sex group of SUD subjects in ambulatory care with total abstinence as treatment goal. This study showed no significant inter-group differences between subjects with and without a history of sexual abuse in terms of rate of abstinence or Addiction Severity Index (ASI) composite scores [24]. These contradictory data may be attributable to the heterogeneity of the respective samples (male/female, drug preferences, in/outpatient settings). Research into specific populations is therefore warranted. The majority of people with AUD in the Swedish population are socially established with housing, education, work and family [25]. However, few studies to date have examined if and how a history of childhood abuse is associated with treatment outcome in socially stable women with AUD,

The aim of the present study was to investigate whether a history of childhood abuse is associated with the degree of change in alcohol consumption in socially stable women undergoing treatment for AUD at an addiction centre focusing on women. On the basis of previous studies, we hypothesised that women with a history of childhood abuse would show less favourable outcomes both immediately after treatment and at 12-month follow-up.

## Methods

The study was performed within the context of the Kajsa Centre which was initiated to motivate socially well-adjusted women with AUD to seek treatment and who due to e.g. fear of stigmatisation, hesitated to seek treatment at a conventional addiction unit. The centre is located in Örebro County (in central Sweden) and offers, to county residents, an outpatient treatment for AUD to women aged ≥25 years without excessive social and/or psychiatric problems. Women at the Kajsa Centre are estimated to be representative for the Swedish general female population with AUD.

The patients are offered individual and/or group sessions once a week. The duration of treatment is individually decided according to each patients wish and need. The treatment is offered as part of the public health care system in Sweden. During the treatment the patients continued with their life with respect to work, family and other social duties as usual.

The overall aim of this study is to increase knowledge concerning personality, psychiatric comorbidity, treatment outcome and quality of life in this population. The present study included women who fulfilled the criteria for alcohol abuse or alcohol dependence according to the Diagnostic and Statistical Manual of Mental Disorders Fourth Edition [26]. Participants with ongoing comorbid illicit substance abuse or dependence were excluded. The study was approved by the Regional Ethical Review Board of Uppsala, Sweden, and all participants provided written informed consent prior to inclusion.

### Study design and procedure

In this longitudinal study the participants were interviewed at the beginning and end of treatment and at 12 month follow-up after end of treatment using the Addiction Severity Index (ASI) [27–29] and the Mini International Neuropsychiatric Interview (MINI) [30, 31]. The self-rating Childhood Trauma Questionnaire (CTQ) [32] was completed at the beginning of treatment. Drinking-related data were collected at the beginning and end of treatment and at 12-month follow-up using the self-rated Alcohol Habits Inventory-Revised 2 (AVI-R-2) [33, 34]. The three outcome measures of change in alcohol consumption were calculated between the start and end of treatment and between start and 12 month follow-up.

### Participants

Participants were recruited between April 2012 and June 2015. All patients who started treatment at the Kajsa Centre and fulfilled the inclusion criteria were asked by staff to participate in the study. Oral and written information was provided about the study. Data collection continued until September 2017. During the recruitment period, 149 participants fulfilled the study inclusion criteria. Of these, 70 participants were not included as they either declined study participation (*n* = 59), dropped out of treatment before the commencement of data collection (n = 5) or were not invited to participate in the study due to an administrative error (*n* = 6). Of the 79 included subjects, four women did not complete the study questionnaires and their data were therefore excluded from the analyses.

An attrition analysis revealed no differences in terms of age (mean (M) and range) between included and non-included individuals. The final sample thus comprised 75 subjects (alcohol dependence, *n* = 69; alcohol abuse, n = 6). The age range of the cohort was 25 to 72 years (M = 50.5, standard deviation (SD) = 11.7). Marital status, educational level and occupational status are shown in Table 1. The majority of the participants were employed and had a stable place of residence. A minority of the cohort (*n* = 7) were retired. The median duration of treatment for the total cohort (*n* = 75) was 8 months [range, 1–36 months].

**Table 1:** Sociodemographic characteristics, parental history of alcohol/substance problems, psychiatric symptoms and alcohol parameters in the study cohort. Data are presented as the mean (SD), median (min–max) or frequency (percent)

	All subjects women n = 75	Childhood abuse *n* = 38	No childhood abuse *n* = 37	*p*-value test statistics
Age, years				
Mean (SD)^a^	50.5 (11.7)	48.9 (12.2)	52.1 (11.1)	*p* = 0.24
Median (min–max)	51.0 (25–72)	48.0 (25–72)	53.0 (25–71)	
Cohabitant				
n (% yes)^b^	38 (50.7%)	17 (44.7%)	21 (56.8%)	*p* = 0.36
School education in years				
Mean (SD)^a^	12.5 (2.6)	12.9 (2.8)	12.1 (2.4)	*p* = 0.18
Median (min–max)	12.0 (8–20)	12.0 (9–20)	12.0 (8–17)	
Employment or studying				
n (% yes)^b^	60 (80.0%)	30 (78.9%)	30 (81.1%)	*p* = 1.00
Maternal alcohol /substance problem				
n (% yes)^b^ *n* = 74	15 (20.3%)	11 (28.9%)	4 (11.1%)	*p* = 0.083
Paternal alcohol /substance problem				
n (% yes)^b^ n = 74	29 (39.2%)	15 (39.5%)	14 (38.9%)	*p* = 1.00
Lifetime depression				
n (% yes)^b^	50 (66.7%)	28 (73.7%)	22 (59.5%)	*p* = 0.23
Lifetime anxiety				
n (% yes)^b^	59 (78.7%)	32 (84.2%)	27 (73.0%)	*p* = 0.27
Lifetime suicide attempt				
n (% yes)^b^	26 (34.7%)	17 (44.7%)	9 (24.3%)	*p* = 0.090
Onset age of AUD				
Mean (SD)^a^	43.2 (12.7)	40.2 (13.8)	46.2 (10.9)	*p* = 0.042
Median (min–max)	44.0 (14–70)	41.0 (14–68)	45.0 (23–70)	
Years of AUD				
Mean (SD)^c^	7.3 (7.7)	8.7 (8.3)	5.9 (6.9)	*p* = 0.106
Median (min–max)	5.0 (0–32)	6.0 (0–32)	3.0 (0–31)	

^a^ Two-sample t-test

^b^ Fisher’s exact test

^c^ Mann-Whitney U-test

Abbreviations: SD, standard deviation; AUD, alcohol use disorder

Of the 75 participants, 59 (79%) completed the measures at the end of treatment and 57 (76%) at 12-month follow-up. No significant differences were found between subjects who completed the study and those who did not in terms of history of childhood abuse, sociodemographic factors (cohabitant, years of school education, employment/studying), psychiatric symptoms (depression, anxiety, suicide attempts), age of AUD onset or grams of pure alcohol/week at the start of treatment. Similarly, no significant differences were found in terms of reported alcohol/substance problems in parents. However, compared with participants who did not complete the measures at end of treatment, the 59 participants who completed the measures at this time-point had a significantly longer duration of treatment (median (Md): 9 months [range, 3–36] vs Md: 4 months [range, 1–24]; *p* = 0.03), were significantly older (52.3 years, SD: 10.9 vs 43.6 years, SD: 12.1; *p* = 0.007) and had a significantly longer duration of AUD (Md: 6.0 years [range, 0–32] vs Md: 2.0 years [range, 0–30]; *p* = 0.037). No significant differences in the aforementioned variables were found between the 57 participants who took part in the 12-month follow-up assessment and those who did not.

### AUD treatment program

All participants were offered standard treatment at the Kajsa Centre. During the initial sessions, a general assessment of the participant was made, with particular focus on alcohol problems. Individual weekly therapy sessions, based on cognitive behavioural methods were offered. The sessions included discussions concerning motivation to change, the function of alcohol in the participants’ lives, relapse prevention and psychosocial problems. If necessary, the participants were offered a period of inpatient detoxification. Each participant determined whether the goal of treatment was to reduce drinking to a low-risk level (i.e., ≤108 g of pure alcohol per week), or abstinence. If a participant who had opted for low-risk drinking experienced difficulties in maintaining low-risk consumption, a period of abstinence was planned. Group sessions on relapse prevention and also pharmacotherapy, based on addiction status and mental health condition, were offered. All therapists were licensed nurses or social workers. During the course of treatment, all participants also underwent a medical and/or psychiatric evaluation by a physician. In addition, the centre offered psychotherapy and/or neuropsychiatric investigation when deemed necessary. Participants who maintained a stable period of abstinence were able to decide whether to remain abstinent or to consume alcohol below the level of risk-drinking.

### Instruments

#### Addiction severity index (ASI)

Data on years of education, employment, marital status, psychiatric symptoms and parental alcohol/substance problems were obtained using the ASI interview [27, 28]. To generate data on parental abuse of prescribed drugs, the parental abuse question was phrased as follows: “Did your father and/or mother have severe problems with alcohol, narcotics and/or prescribed drugs”. The responses were dichotomised to yes or no alcohol/substance problems.

#### MINI international neuropsychiatric interview (MINI)

Psychiatric and alcohol/substance use disorders were diagnosed using the structured diagnostic interview MINI, in accordance with DSM-IV criteria*.* MINI was designed to generate a rapid and reliable diagnosis, and has shown a very good inter-rater and test-retest reliability and a high correlation with the Structured Clinical Interview for DSM Disorders and the Composite International Diagnostic Interview [30, 31]. For the purposes of the present study, only the AUD and SUD domains were used. Age of onset for AUD was defined as the age at which the participant first fulfilled DSM-IV criteria for AUD.

#### Childhood trauma questionnaire-short form (CTQ-SF)

The CTQ-SF self-report questionnaire comprises 28 statements and measures five forms of childhood trauma: emotional abuse, physical abuse, sexual abuse, emotional neglect and physical neglect. The questionnaire has been validated for the identification of early childhood trauma [32]. Each statement is answered according to a five-point Likert scale ranging from 1 (never true) to 5 (very often true). Scores for each subscale range from 5 to 25. Higher scores indicate a greater severity of abuse. Due to both the more severe impact of abuse compared with neglect, and the lack of influence of neglect in a previous study [14], only the three subscales of abuse (emotional, physical and sexual) were used in the present study. A detailed description of these three subscales is presented elsewhere [14]. For the purposes of the present analyses, childhood abuse was defined as a childhood history of 1–3 forms of abuse, namely, emotional, physical and/or sexual abuse, according to the CTQ-SF.

#### Alcohol use assessment

The three outcomes of interest were as follows: changes in alcohol consumption (grams of pure alcohol/week); risk-drinking; and reported abstinence. Intra- and inter-group comparisons were made at the end of treatment and at 12-month follow-up.

Alcohol consumption was assessed using the Alcohol Habits Inventory-Revised 2 (AVI-R-2) form. The AVI-R-2 measures subjective causes of drinking and alcohol-related complications [33, 34].. Items addressing high consumption in AVI-R-2 are based on the three consumption questions of the Alcohol Use Disorders Identification Test (AUDIT-C) [35]. For the purposes of the present study, only two of the questions for high consumption were used. The first question is “How often do you have a drink containing alcohol?” with the alternative answers never, monthly or less, 2–4 times a month, 2–3 times a week or four or more times a week. The second question is “How many drinks containing alcohol do you have on a typical day when you are drinking?” with the alternative answers one or two, three or four, five or six, seven or nine drinks, two bottles of wine or 37 cl spirits, four bottles of wine or one bottle of spirits or more. In accordance with the criteria of the Swedish National Board of Health and Welfare, the alcohol content of a standard drink was defined as 12 g of pure alcohol. Responses were presented as a mean value (e.g., 1–2 standard drinks were recorded as 1.5 standard drinks). The weekly consumption of alcohol was then calculated. In Sweden, risk-drinking for women is defined as > 108 g of pure alcohol per/week [36]. The results were dichotomised as non-risk drinking = 0 and risk-drinking = 1. Abstinence was defined as not using alcohol at all.

#### Statistics

With a sample size of 27 patients in each group the study had a power of 88.7% if % improvement in abstinence is 13% in the group with childhood abuse and 56% in the group without childhood abuse, with Fisher’s exact test on significant level 0.05, two sided test.

The distribution of variables was described in terms of the mean and SD. To increase information on the distribution of data, for continuous variables the median and variation (min–max) were also used. Categorical variables were described in terms of numbers and percentages.

For inter-group (abuse/no abuse) comparisons of sociodemographic, parental, psychiatric and alcohol variables at the start of treatment, the following tests were used: two-sample t-test for normally distributed values; the Mann-Whitney U-test for non-normal continuous variables; and Fisher’s exact test for dichotomous variables.

For intra-group comparisons of the change in alcohol consumption between two evaluation time-points (from start of treatment to end of treatment and from start of treatment to 12-month follow-up), the Wilcoxon signed rank test was used for continuous variables, and the Exact McNemar’s test was used for ordered categorical variables (categorised as worse, equal or improved).

For inter-group comparisons of the change in alcohol consumption, the Mann-Whitney U-test was used for continuous variables and the Mantel-Haenszel Chi square test was used for ordered categorical variables. For dichotomised variables, Fishers exact test was used. All significance tests were two-sided and conducted at the 5% significance level.

## Results

### Frequency of childhood abuse

A total of 50.7% of the cohort reported childhood abuse (emotional, physical and/or sexual). The most commonly reported form of abuse was emotional abuse (40.0%), followed by physical abuse (37.3%) and sexual abuse (18.7%). In total, 28.0% of the cohort reported more than one form of abuse. Overall, the proportions of participants who met the threshold for 1, 2 or 3 forms of abuse, respectively, were 22.7% (*n* = 17), 10.7% (*n* = 8) and 17.3% (*n* = 13).

### Background variables

No significant inter-group differences were found for cohabitation, employment, years of education, psychiatric symptoms or parental alcohol/substance problems. With regard to alcohol variables at the start of treatment, participants with a history of childhood abuse had an earlier age of AUD onset (t = 2.069; *p* = 0.042; see Table 1). However, no significant inter-group differences were found in terms of AUD duration or alcohol consumption (grams of pure alcohol per week) (Mann-Whitney U-test; *p* = 0.87).

### Outcomes at the end of treatment

No significant inter-group difference was found for treatment duration (Mann-Whitney U-test; *p* = 0.73). At the end of treatment, both groups showed significant improvements in terms of grams of pure alcohol per week and risk-drinking (see Table 2). In total, 10.7% of the non-abused group maintained risk-drinking compared with 32.3% in the abused group (Fisher’s exact test, *p* = 0.062). However, a significant inter-group difference was found regarding abstinence. Here, the non-abused group included a significantly higher proportion of participants with abstinence (39.3%) than did the abused group (12.9%) (Fisher’s exact test, *p* = 0.035), see Table 2.

**Table 2:** Intra- and inter-group comparisons of change in alcohol consumption between the start and end of treatment and at 12 month follow-up, as measured according to three outcome measures^1^

	Childhood abuse			No childhood abuse			Inter-group comparison	
	Start	Change at end of treatment	Change at 12-mo F/U	Start	Change at end of treatment	Change at 12-mo F/U	End of treatment	12-mo F/U
	n = 38	*n* = 31	*n* = 30	n = 37	*n* = 28	*n* = 27	n = 59	n = 57
Grams/w^1^								
M (SD)	299 (290)	− 143 (132)	− 112 (128)	327 (355)	− 240 (301)	− 357 (403)		
Md (min–max)	173 (32–1584)	−132 (− 423 − + 51)	−91 (− 363 − + 165)	231 (14–1584)	− 159 (−1584–0)	−240 (−1584 - -45)		
*p*-value		*p* < 0.001^a^	*p* < 0.001^a^		*p* < 0.001^a^	*p* < 0.001^a^	*p* = 0.174^c^	*p* = 0.001^c^
Risk-drinking^1^								
n (%)	28 (73.7%)			28 (75.7%)				
Worse n (%)		0 (0%)	1 (3.3%)		0 (0%)	0 (0%)		
Equal n (%)		18 (58.1%)	17 (56.7%)		10 (35.7%)	5 (18.5%)		
Improved n (%)		13 (41.9%)	12 (40.0%)		18 (64.3%)	22 (81.5%)		
*p*-value		*p* < 0.001^b^	*p* = 0.003^b^		*p* < 0.001^b^	*p* < 0.001^b^	*p* = 0.089^d^	*p* = 0.002^d^
Abstinence^1^								
n (%)	0 (0.0%)			0 (0.0%)				
Worse n (%)		0 (0%)	0 (0%)		0 (0%)	0 (0%)		
Equal n (%)		27 (87.1%)	26 (86.7%)		17 (60.7%)	12 (44.4%)		
Improved n (%)		4 (12.9%)	4 (13.3%)		11 (39.3%)	15 (55.6%)		
*p*-value		*p* = 0.125^b^	*p* = 0.125^b^		*p* = 0.001^b^	*p* < 0.001^b^	*p* = 0.021^d^	*p* = 0.001^d^

^1^Outcome measures were:

Grams/w: Grams of pure alcohol per week in M (SD) and Md (min–max),

Risk-drinking: alcohol consumption > 108 g of pure alcohol per week, n (%), categorised as worse, equal or improved compared with start,

Abstinence: Not using alcohol at all n (%)

Abbreviations: M: mean, SD: standard deviation, Md: median, min-max: minimum to maximum, n: number in subgroup, N: total number in sample, mo F/U: month follow-up

Statistic test used: ^a^ Wilcoxon signed rank test, ^b^ Exact McNemar’s test, ^c^ Mann-Whitney U-test, ^d^ Mantel-Haenszel Chi square test

### Outcomes at 12-month follow-up

At 12-month follow-up, both groups showed a statistically significant decrease in alcohol consumption compared with the start of treatment. However, the change in grams of pure alcohol per week was significantly higher in the non-abused group (Mann-Whitney U-test, *p* = 0.001). In terms of risk-drinking, a significant improvement was found in both groups. However, the improvement in the non-abused group was significantly more pronounced (χ^2^ = 10.055, *p* < 0.01). Around 82% of participants in the non-abused group showed an improvement, compared with 40% in the abused group. At 12-month follow-up, no risk-drinking at all was reported in the non-abused group, compared with 36.7% in the abused group (Fisher’s exact test; *p* < 0.001). A significant inter-group difference was also found for abstinence, with abstinence being reported in 55.6 and 13.3% of participants in the non-abused and abused groups, respectively (Fisher’s exact test, *p* = 0.002), see also Table 2.

## Discussion

The present study examined the association between childhood abuse and the degree of change in alcohol consumption from start to end of treatment and at 12-month follow-up in a cohort of socially stable women with AUD.

The main finding was a more favourable treatment outcome in participants with no history of childhood abuse. At 12-month follow-up, a history of childhood abuse was significantly associated with less positive results in all treatment outcomes. For example, a significantly higher proportion of non-abused participants achieved abstinence (55.6% vs 13.3%). This supports the study hypothesis of less favourable outcomes in abused women at 12-month follow-up. The hypothesis concerning change of alcohol consumption at the end of treatment was only partly confirmed, since both groups showed a significant improvement during treatment, with no significant inter-group differences in terms of grams of pure alcohol per week or risk-drinking. However, the non-abused group included a significantly higher proportion of participants with reported abstinence than did the abused group (39.3% vs 12.9%).

At the end of treatment and at 12-month follow-up, a significant reduction in alcohol consumption was observed in both groups. This finding is consistent with previous reports [22–24, 37].

The findings of the present analyses also support several previous investigations of AUD and SUD participants with a history of childhood abuse, which reported less response to treatment, shorter time to first drink after treatment and a higher risk of relapse [21, 22, 37]. By contrast, other studies have found similar improvements in abused and non-abused participants in terms of ASI alcohol and drug composite scores at follow-up [23, 24]. However, all but one [21] of the aforementioned studies included participants with predominant drug abuse [22–24, 37], and only one [22] concerned women only. The present findings may be attributable to the use of a more homogenous cohort, which comprised socially stable, educated women with high rates of partnership and employment.

The present data suggest that, while women with a history of childhood abuse may improve and maintain their improvement in alcohol consumption during the course of treatment, they may experience difficulties in maintaining these effects once treatment is complete. By contrast, non-abused women may be more likely to maintain or continue their improvement post-treatment. The inter-group difference in the proportion of abstainers was substantial. Abstinence from alcohol at the end of treatment represents the best drinking-related outcome for individuals with AUD, and is associated with the best long-term outcome [38–40]. Further research is warranted to determine whether individuals with a history of childhood abuse are psychologically prepared, and have the ability, to accept abstinence.

The prevalence of the different forms of childhood abuse (emotional, physical and sexual) in the present cohort was consistent with that found in previous studies of AUD in mixed-sex populations [15], as well as in studies that focused on women only or that stratified their analyses according to gender [7, 8, 12, 13]. However, a degree of cross-study variation in the prevalence of the different forms of childhood abuse is evident. This may be attributable to the nature of the study populations but also to the study design and the varying ways of obtaining information about childhood abuse.

In contrast to all but one [22] previous study of treatment outcome in AUD and SUD, the present study also included participants with childhood emotional abuse. The reason for this was to take into account, recent findings on the importance of emotional abuse in the development and severity of AUD [8, 15]. In total, 40% of the participants reported a history of childhood emotional abuse. Among others, childhood emotional abuse may result in difficulties in interpersonal relationships, for example, a craving for (or avoidance of) attention, inattention and difficulties in the regulation of emotion and temper [41]. A history of childhood emotional abuse may therefore impact on treatment outcome.

### Strengths and limitations

A strength of the present study was the use of a fairly homogenous group of adult women with AUD. Earlier studies of childhood abuse and treatment outcome used mixed-sex samples of participants with combined alcohol and drug abuse, which might have impacted their results and rendered them more difficult to interpret [21–24]. A further strength of the present study was the use of a cohort of educated and socially stable women with high rates of partnership and employment. We believe that the specific approach of the Kajsa Centre enables more women with socially stable conditions to seek treatment for AUD and thus describes adequately this group of patients. It is however not possible to generalize our results to women with comorbid illicit drug abuse and/or severe psychiatric comorbidity.

A limitation of the present study was that childhood abuse and alcohol consumption were measured subjectively using self-report instruments that may have been subject to recall bias. However, all questionnaires used in the study are well-established in the field.

A further limitation was the relatively small sample size, which was exacerbated by drop-out rates of 21% at the end of treatment and 24% at 12-month follow-up. However, this degree of drop-out is usual in clinical studies, and the attrition analysis at 12-month follow-up revealed no differences between participating and non-participating participants. In addition, the power analysis suggested that the sample was of sufficient size.

## Conclusions

The present study examined the relationship between a history of childhood abuse and treatment outcome, as measured according to alcohol consumption at the end of treatment and at 12-month follow-up, in socially stable women with AUD. At 12-month follow-up, women with a history of childhood abuse showed less improvement in all outcome variables than women with no history of abuse. Women with a reported history of childhood abuse had more difficulties in maintaining their improvement in alcohol consumption after treatment than did non-abused women. A plausible hypothesis is that women with a history of childhood abuse may have been less psychologically prepared to abstain from alcohol.

The present findings highlight the importance of identifying a history of childhood abuse in socially stable women seeking treatment for AUD. In terms of clinical practice, the results suggest that, to achieve sustained and long-term effects, additional treatment and more extended follow-up may be necessary in women with a history of childhood abuse. These patients might be in need of psychotherapeutic interventions that focuses on their traumatic experiences, in parallel with treatment for AUD. This needs to be further examined. Future research should investigate the different types and severity of childhood abuse and how they affect treatment outcome. There is also a need to involve larger samples and biological markers of alcohol consumption.

## Data Availability

The datasets used and analysed during the current study are available from the corresponding author on reasonable request.

## Abbreviations

ASI: Addiction Severity Index
AUD: Alcohol use disorder
AUDIT-C: Alcohol Use Disorder Identification Test
AVI-R-2: Alcohol Habits Inventory-Revised 2
CTQ-SF: Childhood Trauma Questionnaire-short form
M: Mean
Md: Median
MINI: Mini International Neuropsychiatric Interview
SD: Standard deviation
SUD: Substance use disorder
